# Human–Tiger Conflict in and Around Chitwan–Parsa National Parks: Status, Trends, Contributing Factors, Mitigation Strategies, and Community Perceptions

**DOI:** 10.1002/ece3.73920

**Published:** 2026-07-15

**Authors:** Aayush Shrestha, Narendra Man Babu Pradhan, Naresh Subedi, Bikram Shrestha, Divesh Shrestha, Mahesh Neupane, Krishna Prasad Dahal

**Affiliations:** ^1^ Institute of Forestry Hetauda Campus Tribhuvan University Hetauda Nepal; ^2^ International Union for Conservation of Nature (IUCN) Kathmandu Nepal; ^3^ National Trust for Nature Conservation (NTNC) Lalitpur, Kumaltar Nepal; ^4^ Conservation Development Foundation Nepal (Codefund) Kathmandu Nepal; ^5^ Department of National Parks and Wildlife Conservation Babarmahal, Kathmandu Nepal

**Keywords:** awareness and training, ecological carrying capacity, hotspot mapping, mitigation measures, rear face‐masks, spatio‐temporal patterns

## Abstract

Human casualties are among the most severe consequences of human–tiger conflict (HTC), and recent evidence from Nepal indicates an increasing trend in tiger‐related incidents. This decade‐long study (2014/15–2023/24 A.D.) examined the status, trends, associated factors, conflict patterns, and mitigation preferences related to HTC in and around Chitwan and Parsa National Parks, including surrounding forests of Parsa, Bara, Makawanpur, Chitwan, and Nawalpur districts. Primary data were collected through semi‐structured interviews with all reported victims or household representatives. Analyses included descriptive statistics, Chi‐squared tests, correlation analysis, Friedman ANOVA, and hotspot mapping using MS Excel, SPSS, ArcGIS, and Python. A total of 80 tiger attacks were recorded, with fatalities and injuries occurring in nearly equal proportions. Most cases occurred in the Chitwan National Park buffer zone, and males comprised more than three‐fourths of victims, largely reflecting their involvement in high‐risk forest‐related activities. Recorded casualties increased during the study period and showed a positive association with tiger population estimates; however, this relationship should be interpreted as associative rather than causal because multiple ecological and human‐use factors may also contribute. Hotspot analysis identified Budhirapti BZUC, Madi Valley, and adjoining areas as major conflict zones. Incidents were more frequent during the monsoon and spring seasons, and solitary individuals were more vulnerable than those in groups. Most attacks involved older, injured, or sub‐adult tigers. Awareness and training were ranked as the most preferred mitigation measures, and local communities generally maintained positive attitudes toward tiger conservation despite increasing conflict risks. The findings highlight the need for targeted, context‐specific mitigation strategies, including awareness and training, strengthened physical barriers, management of problem tigers, and further testing of rear‐face masks to support safer human–tiger coexistence.

## Graphical abstract

This decade‐long study examined 80 reported human casualty cases from human–tiger conflict in and around the Chitwan–Parsa National Parks, Nepal. Human casualties increased over the study period and were positively associated with tiger population recovery, although this relationship should be interpreted as associative rather than causal. Hotspot mapping, incident analysis, and community responses indicate the need for targeted mitigation, including awareness and training, strengthened barriers, and management of problem tigers to support safer coexistence.

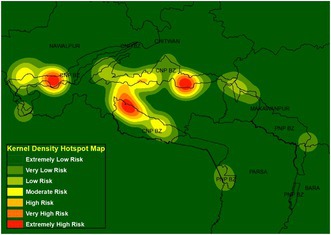

## 1 Introduction

Human–wildlife conflict (HWC) represents an escalating conservation and social challenge driven by expanding human populations, habitat fragmentation, and growing overlap between human settlements and wildlife habitats. Human–tiger conflict (HTC) is particularly severe because it threatens human safety and livelihoods and can trigger retaliatory killings that undermine tiger conservation (Nyhus and Tilson 2004; Inskip and Zimmermann 2009). Incidents typically involve human injury or mortality and livestock depredation, thereby intensifying tensions between conservation goals and community well‐being.

Globally, wild tiger numbers have declined by 95% over the last century from an estimated 100,000 individuals (Wolf and Ripple 2017), leading to the species' “Endangered” status on the IUCN Red List (Goodrich et al. 2022). In 2010, the Global Tiger Recovery Program (GTRP) set an ambitious target to double wild tiger numbers by 2022. Nepal, one of 13 range countries, committed to increase its tiger population from 121 in 2010 to 250 by 2022 and ultimately surpassed this goal, reaching 355 in 2022 (GTRP 2010; DNPWC and DFSC 2022). Tigers in Nepal primarily occupy the lowland Terai within Protected Areas (PAs) such as Chitwan National Park (CNP), Parsa National Park (PNP), Bardiya National Park (BNP), Banke National Park (BaNP), and Shuklaphanta National Park (ShNP), with occasional records above 3100 m (Bista et al. 2021; WWF 2021). CNP and PNP have shown substantial tiger growth—CNP from 91 (2009) to 128 (2022) and PNP from 4 to 41 over the same period (DNPWC and DFSC 2022). While this recovery is a major conservation achievement, it heightens the imperative to balance species protection with community safety, particularly as ecological carrying capacity (ECC) may be approached in some parks (DNPWC 2020; DNPWC and DFSC 2022).

With Nepal's increasing tiger population, HTC incidents including attacks on humans and livestock have risen across PAs (Mandal 2021; Rauniyar 2021; Pokharel 2022; Paudel et al. 2024). Between 2000 and 2020, Nepal recorded 1139 cases of wildlife mortality and 887 cases of human mortality by wildlife (Baral et al. 2022). In CNP, tigers are among the leading causes of wildlife attacks on humans with 70 cases in 2009–2020 (Kandel et al. 2023), while BNP and BaNP documented 34 tiger‐related fatalities from 2019 to 2022 (Kadariya et al. 2023) causing up to 75% of attacks to humans only in recent times (Paudel et al. 2024). Long‐term series from CNP show increasing HTC through time (Gurung et al. 2008; Silwal et al. 2017; Dhungana et al. 2018; Lamichhane et al. 2018). Tigers and leopards have caused > 90% of livestock depredation in CNP (2012–2016) (Lamichhane et al. 2018) and similarly high proportions in PNP (2012–2018) (Parajuli 2020), and multiple retaliatory killings of tigers have been recorded in CNP (Dhungana et al. 2018; Lamichhane et al. 2017; Bhattarai et al. 2019). Although the government has initiated programs to identify and respond promptly to problem tigers, these patterns still underscore the urgency of targeted, evidence‐based risk reduction.

Previous studies have identified key correlates of HTC, including frequent incidents during natural resource collection, elevated risk when people travel alone, and the involvement of older, injured, or dispersing tigers. (Sunquist 1981; Smith 1993; Corbett 2005; Nyhus and Tilson 2010; Dhanwatey et al. 2013; Lamichhane et al. 2017; Kolipaka et al. 2017). Yet the evidence is short‐term or site‐specific and may not integrate landscape‐scale spatio‐temporal analyses with community preferences for mitigation. Consequently, managers still lack decadal, decision‐ready evidence on when and where HTC concentrates, which human situations and tiger traits elevate risk and severity, and which interventions local people consider acceptable and effective across the Terai Arc Landscape (TAL). Mapping conflict hotspots, pinpointing behaviorally high‐risk tigers, and identifying ecological triggers at the TAL scale therefore remain underexplored.

Despite ongoing mitigation practices such as electric fencing, trenches, predator‐proof corrals, physical barriers, regular patrolling, improved compensation schemes, and awareness and training programs (Inskip and Zimmermann 2009; Goodrich 2010; Dhungana et al. 2024), effectiveness remains context‐dependent. In some places, communities continue to support tiger conservation even under high conflict, whereas elsewhere retaliatory threats persist, often linked to perceived shortcomings in park response, delays or inadequacy in compensation, and limited uptake of non‐lethal options (Mukherjee 2003; Barlow et al. 2010). These mixed outcomes underscore the need to pair spatio‐temporal risk evidence with community‐endorsed measures so that management actions are both targeted and socially acceptable.

While previous studies in Nepal and the TAL have largely been short‐term or focused on isolated factors of HTC, long‐term and integrative assessments remain limited. Here, we present a decadal (2014/15–2023/24) dataset based on complete enumeration of human casualty cases from the Chitwan–Parsa complex, one of the few such datasets available for Nepal, covering all 80 recorded human casualty incidents across both CNP and PNP. Unlike earlier studies, our analysis simultaneously integrates spatio‐temporal patterns, human behavioral factors, tiger characteristics, and community perceptions within a unified framework. We specifically examined (i) temporal trends, (ii) spatial concentration of attacks relative to forest edges and human‐use areas, (iii) the influence of human activities and tiger traits on conflict occurrence and severity, and (iv) community preferences for mitigation strategies ranked by local acceptability. By linking these dimensions with the broader context of tiger population recovery, this study provides landscape‐level evidence relevant to adaptive management of human–tiger coexistence in the TAL.

## 2 Methods

### 2.1 Study Area

**FIGURE 1:**
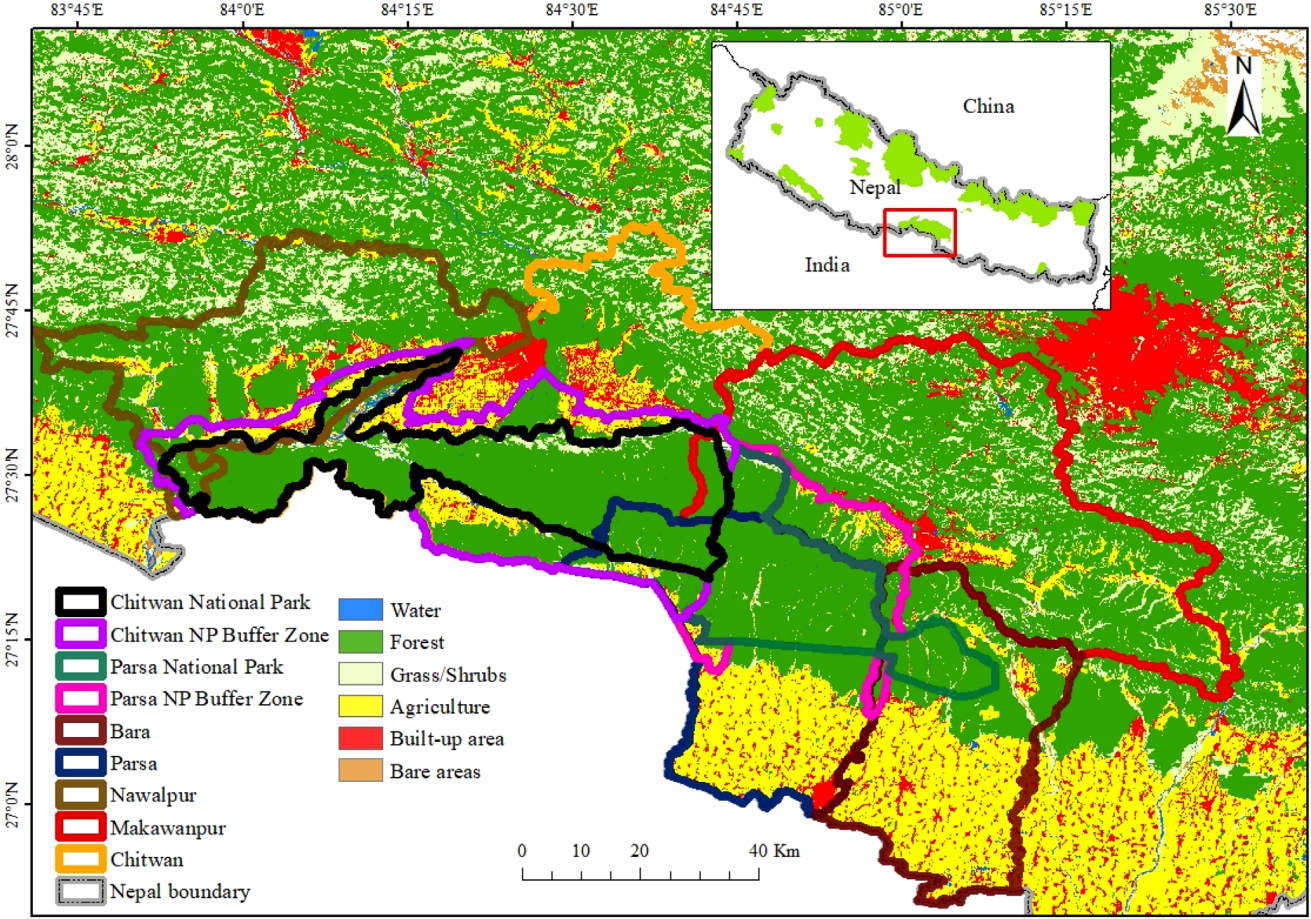
Location map of the study area, that is, BZs of CNP, PNP and adjoining forests.

The study area includes the Buffer Zones (BZs) of CNP and PNP, along with adjoining forests in Chitwan, Bara, Parsa, Makawanpur, and Nawalpur districts (Figure 1). CNP, located in the subtropical Inner Terai lowlands of south‐central Nepal, spans 952.63 km^2^ and is bordered by the Narayani and Rapti rivers, the Churia Hills, and the Mahabharat Range. Its diverse habitats include sal forests, riverine forests, grasslands, and wetlands, supporting rich biodiversity with 70 mammal species, over 600 bird species, and 56 reptiles and amphibian species (Dhungana et al. 2018). CNP experiences a monsoon‐dominated subtropical climate with annual rainfall of 2250 mm and temperatures ranging from 11°C to 38°C.

PNP, located in the Parsa, Makwanpur, and Bara districts near the Indian border, covers 627.4 km^2^ and is known for its diverse flora and fauna, including Bengal tigers ( *Panthera tigris tigris* ), Gaur ( *Bos gaurus* ), and residential wild elephants ( *Elephas maximus* ), and various bird species. It features tropical and subtropical forests, grasslands, riverine ecosystems, and wetlands. Its elevation ranges from 100 to 950 m, encompassing the Churia range, Bhabar‐Terai zone, and Inner‐Terai region (DNPWC 2019). PNP is part of the TAL initiative, a vital wildlife corridor connecting Nepal and India. The other area connected to the PAs comprises five districts, that is, Bara, Parsa, Makawanpur, Chitwan, and Nawalpur. Most of the connected forest areas lie in the lowlands of Nepal except the hilly region of Makawanpur, Chitwan, and Nawalpur district.

### 2.2 Data Collection

The research data were collected through primary and secondary sources. Data from the last 10 years about the HTC resulting in human casualties was taken from the office records, annual records, online news, and compensation lists of respective PAs and District Forest Offices (DFOs). To address any potential gaps in official records and gather information on any known incidents of human casualties, we collected data from almost every chairperson or, in their absence, the secretaries of the Buffer Zone User Committees (BZUC) of both CNP and PNP.

Primary data were collected in August–September 2024 through a total enumeration of all reported HTC cases spanning 2071–2080 B.S. (2014/15–2023/24 A.D.). We conducted semi‐structured interviews with survivors for injury cases and with household representatives for fatal cases; when in‐person visits were not feasible, we conducted phone interviews. Interviews lasted approximately 30 min, were conducted in Nepali, Tharu, or Maithili, and were carried out after verbal consent was obtained. Using a standard incident form, we recorded the variables for every case such as victim demographics (age, sex, occupation, education); event date; location (BZUC, ward, attack site); distance to the forest edge or settlement (Euclidean distance calculated from Google Earth Pro); time of day; activity at time of encounter (e.g., fodder/fuel wood collection, agriculture, herding, travel); group size (alone vs. with others); tiger traits as reported by survivors/witnesses or capture teams (age class, sex if known, injury status, prior designation as a “problem” tiger); encounter type (ambush/predation attempt, provoked/retaliatory, accidental) and post‐event responses (medical care sought, compensation claim initiation/decision). We also noted awareness cues (e.g., tracks, warnings) and perceptions of mitigation options. Mitigation measures were regular patrolling, strengthening physical barriers, awareness and training, relocating or taming problematic tigers, killing problematic tigers, and improved compensation schemes. Regular patrolling involved joint teams of park and army personnel patrolling park boundaries. Physical barriers included electric or solar fences, RCC walls, and barbed wire fencing. Awareness programs meant educating people about tiger behavior and ecology, recent increase in tiger population, reason of their growth and their sustainability, ECC, causes of HTC, problematic tigers and mitigation measures. Training refers to teaching efficient ways to be protected in close encounters and ways to avoid attacks. Compensation referred to government aided financial relief for HTC related damages. Relocating or taming problematic animals refers to relocating such problematic animals into other areas of the park, the zoo or animal enclosure in the park. To complement case interviews with data triangulation, we conducted key‐informant interviews with park managers, BZUC representatives, local officials, citizen scientists, and forest guards.

### 2.3 Data Analysis

Data were initially entered into MS Excel and later sorted to IBM SPSS Statistics 29 for analysis. MS Excel was used for trend analysis, and the chi‐square tests assessed the significance of variable frequencies. Kernel density estimation (KDE) was performed in ArcGIS 10.4.1 (ESRI, Redlands, CA, USA) using geographic coordinates of conflict incidents to generate a continuous density surface. A search radius of 6000 m and a cell size of 100 m were applied to capture landscape‐level clustering. The output was classified into seven density categories (extremely low to extremely high) using the natural breaks (Jenks) method. The resulting map was used to identify conflict hotspots and interpret spatial risk patterns in the Chitwan‐Parsa Complex. As some data showed a non‐linear increase, with a sharper rise in the later years of the study period, a second‐degree polynomial fit was used as it had a higher *R* ^2^ value. Data visualization, that is, bar‐graph and correlation was done using Python 3.13. The Friedman ANOVA test was used to evaluate the average mean score of mitigation measures.

To analyze the relationship between tiger population and human casualties, we used tiger population data from national censuses, which were plotted against average annual human casualties in the study area. Pearson's correlation analyzed the relationship between human casualties (2014/15–2023/24) and incidents within 500 m of settlements and seasonal trends. Seasons were classified as follows: spring (mid‐March to May), summer/monsoon (mid‐June to September), autumn (mid‐October to November), and winter (mid‐December to February). In some cases, multiple injuries from a single event were counted as one occurrence to prevent skewed results. For example, in 2016, seven individuals were injured during a group search for a tiger in Nandabhauju BZUC, and in 2018, a man was injured while observing a man‐eating tiger being captured after it killed a woman. Our analytical approach was intentionally descriptive and management‐oriented, appropriate to the nature and scope of the data. Because this study constitutes a complete enumeration of all 80 reported human casualty cases from tiger attacks in the Chitwan–Parsa complex during 2014/15–2023/24, rather than a random sample drawn from a larger population, the primary aim was to characterize the full universe of known incidents across space, time, victim attributes, human activities, encounter conditions, tiger‐related traits, and community‐preferred mitigation strategies. Inferential multivariate modeling is most suited to sampled data where generalization beyond the sample is the objective; for complete enumeration datasets of this kind, comprehensive descriptive characterization constitutes the appropriate analytical framework (Agresti 2002; Hosmer and Lemeshow 2000). We applied inferential tests such as chi‐square, Pearson's correlation, and Friedman ANOVA where appropriate to support pattern identification and assess association strength, interpreting all findings as associations rather than causal evidence, consistent with the observational and retrospective nature of the data.

Tiger characteristics were documented through multiple sources, including group encounters where victims could verify the tiger's traits, survivors who identified the tiger post‐attack, and technical teams involved in capturing or relocating the tiger. In some cases, local observations helped infer the tiger's characteristics. If identification was not possible, the case was recorded as “don't know.” To assess victims' awareness of the conflict risk, we evaluated whether they had detected signs of a tiger's presence, received warnings, or were informed through other means.

Mitigation measures recommended by respondents were categorized into six groups, adapted from Goodrich (2010) and site‐specific strategies from CNP (Dhungana et al. 2024) including the preventative, mitigative, and reactive measures (Goodrich 2010) and were analyzed using Friedman ANOVA.

### 2.4 Limitations of the Study

This study may have some limitations. Although it is based on the total enumeration of all reported human casualty cases from tiger attacks in the study landscape, some incidents may still be unreported. Retrospective interviews may be subject to recall bias, particularly for older cases. Likewise, official records, compensation files, and media reports may vary in completeness and may contain reporting bias or inconsistencies. We reduced these issues by cross‐checking and triangulating incidents across multiple sources wherever possible. Because exposure‐related variables such as human population change, land‐use dynamics, and forest‐use intensity were not consistently available at the case level, some findings are best interpreted as descriptive and management‐oriented rather than causal.

## 3 Results

### 3.1 Status and Trends of Human Casualties in Tiger Attacks

Our data revealed a total of 80 human casualty cases from HTC in the study area: BZ of CNP (*n* = 59, 73.8%), PNP (*n* = 5, 6.4%) and adjoining forest areas comprising Bara (*n* = 2, 2.6%), Parsa (*n* = 1, 1.3%), Makawanpur (*n* = 3, 3.8%), Chitwan (*n* = 2, 2.5%) and Nawalpur (*n* = 8, 10%) in the 2014/15–2023/24 year (Table 1). Almost equal cases resulted in fatalities and injuries (Table 2). There was a significant difference by sex, with male casualties being higher (*χ* ^2^ = 22.05, df = 1, *p* < 0.001). The average age of the victims was 45.31 years (17–69 years). Among the occupation of the victims, farmers were significantly more than the others (*χ* ^2^ = 92.2, df = 6, *p* < 0.001). The significant number of victims had little or no formal education (*χ* ^2^ = 41, df = 3, *p* < 0.001). The socio‐demographic characteristics of the victims are presented in Table 2.

**TABLE 1:** Status of human casualties in BZs and connected forest areas.

Year	CNP BZ		PNP BZ		Bara		Parsa		Makawanpur		Chitwan		Nawalpur	
	D	I	D	I	D	I	D	I	D	I	D	I	D	I
2014/15	3	1	—	—	—	—	—	—	—	—	—	—	—	—
2015/16	1	8	—	—	—	—	—	—	—	—	—	—	—	—
2016/17	2	—	—	1	—	—	—	—	—	—	—	—	—	—
2017/18	—	2	1	1	—	—	—	—	—	—	—	—	—	—
2018/19	3	—	—	—	—	—	—	—	—	—	—	—	—	—
2019/20	2	1	—	—	—	—	—	—	—	—	—	—	—	—
2020/21	3	1	—	—	—	—	—	—	—	—	—	—	1	1
2021/22	6	5	—	—	1	—	—	—	—	—	—	1	2	1
2022/23	6	3	—	—	—	—	—	—	—	1	—	—	—	—
2023/24	3	10	2	—	—	1	1	—	—	2	—	—	2	1
Total	29	30	3	2	1	1	1			3		2	5	3
Grand Total	59		5		2		1		3		2		8	

Abbreviations: D, death; I, Injury.

**TABLE 2:** Socio‐demographic status of the victims from HTC in the study area.

	Total	80 (*n*)	100% (%)
Age	Range = 17–69, mean = 45.31, SD = 13.19		
Type of incident	Death	39	48.8
	Injury	41	51.2
Sex of the victim	Male	61	76.3
	Female	19	23.7
Occupation of the victim	Farming	39	48.8
	Animal husbandry	6	7.5
	Private business	9	11.3
	Govt. Job	2	2.5
	Fishing	1	1.3
	Household works	17	21.3
	Others	6	7.5
Education level of the victim	Illiterate	39	48.8
	Primary	27	33.8
	Lower secondary	14	16.3
	Upper secondary	1	1.3
	Total	80	100%

**FIGURE 2:**
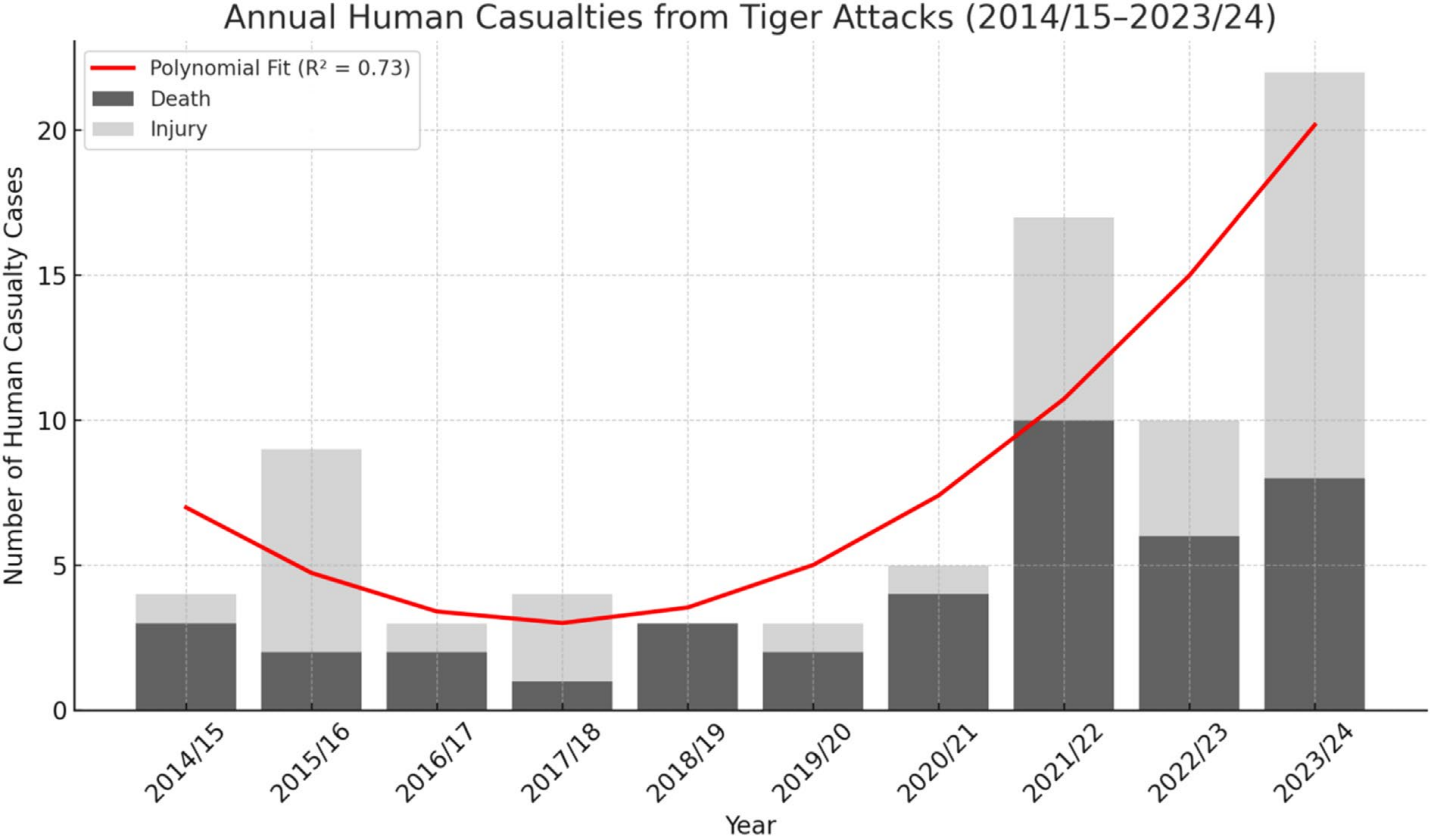
Status and trend of human casualty cases during 2014/15–2023/24 A.D.

Status and trends of annual human casualties caused by HTC between 2014/15 and 2023/24 A.D is presented in the Figure 2 where it shows a significant increase in the study area (*p* < 0.05). The trend analysis with second‐degree polynomial fit (*y* = 0.4659*x* ^2^ – 3.6583*x* + 10.183, *R* ^2^ = 0.73, Figure 2) revealed that HTC cases have been increasing at an increasing rate in recent years with a significant rise in the average number of cases, from 4.6 human casualties per year during 2014/15–2018/19 to 11.4 per year in the period 2019/20–2023/24 A.D.

**FIGURE 3:**
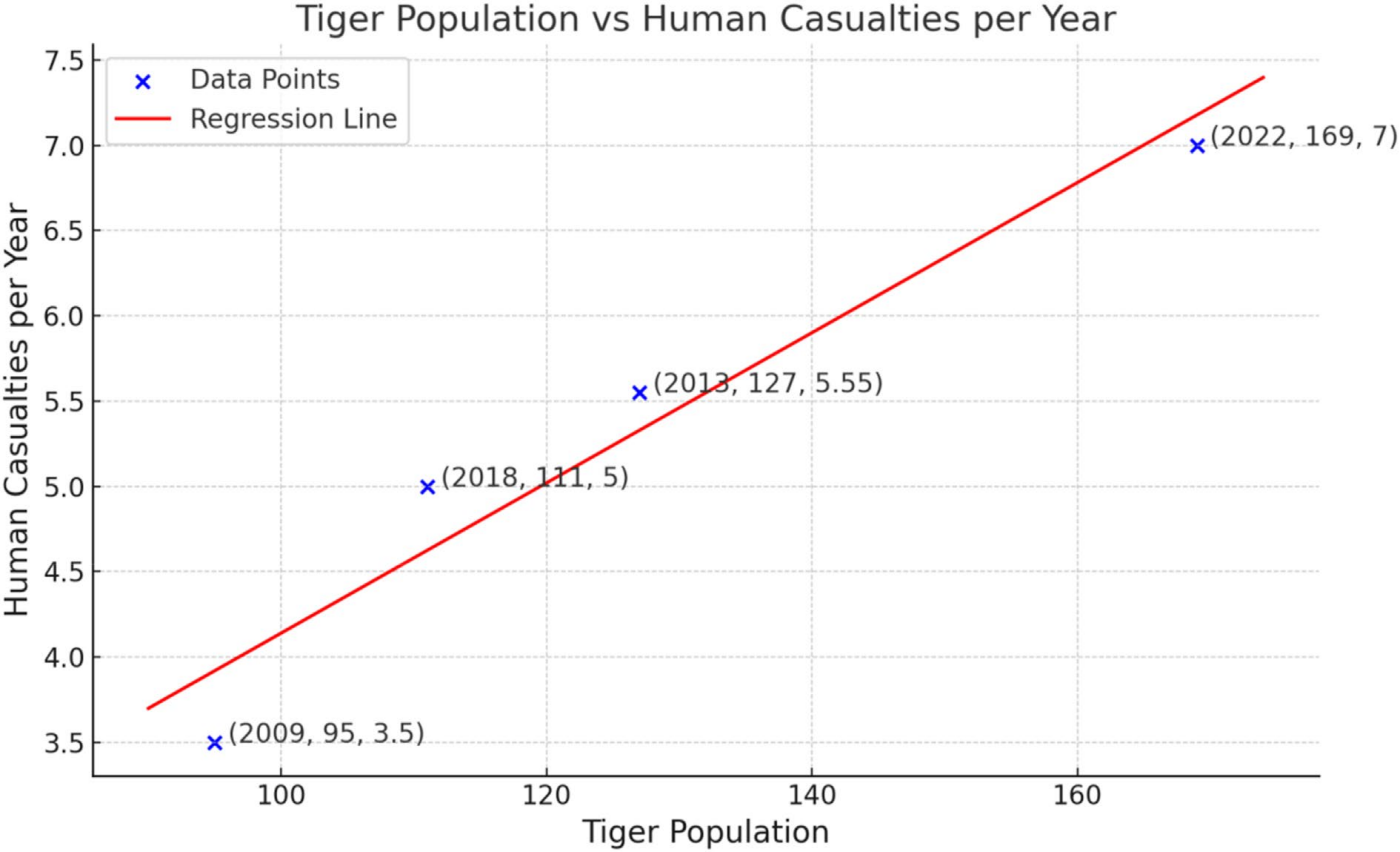
Tiger population versus human casualties (summated avg. per year 2009–2022) in the study area.

We examined the relationship between the increasing tiger population and the corresponding rise in human casualty cases over the period (2009–2022). Human casualty data for the years 2009–2013 were derived from secondary sources, while primary data were collected for the years 2014–2022 within the study area. The annual summated average of human casualties was plotted against tiger population estimates obtained from census year data to assess their relationship. A Pearson correlation analysis showed a positive association between available tiger census estimates and recorded human casualties (*R* ^2^ = 0.90, *p* = 0.03). However, given the limited number of data points (*n* = 4 census years), this relationship should be interpreted cautiously as indicative rather than conclusive and may reflect multiple concurrent factors, including human population growth, settlement expansion, and increasing forest‐resource dependence. Tiger population estimates were obtained from the 2009, 2013, 2018, and 2022 census data where it showed 95, 127, 111, and 169 tigers respectively in the study area. The human casualties per year were 3.5, 5.5, 5, and 7 cases per year respectively for the period between census years (Figure 3).

### 3.2 Factors Associated With Tiger Attacks

**FIGURE 4:**
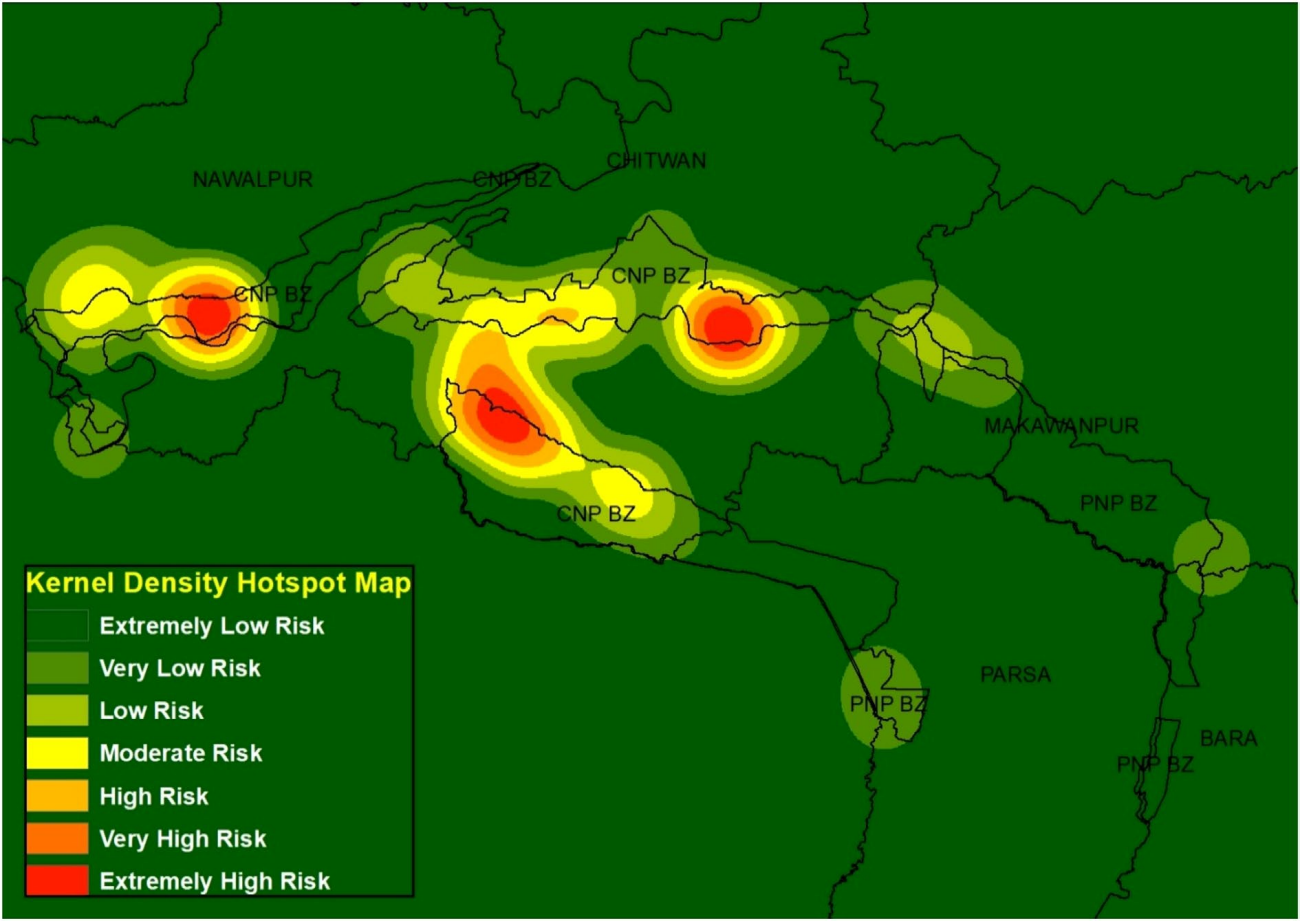
Kernel density hotspot map of human casualties in the study area.

**FIGURE 5:**
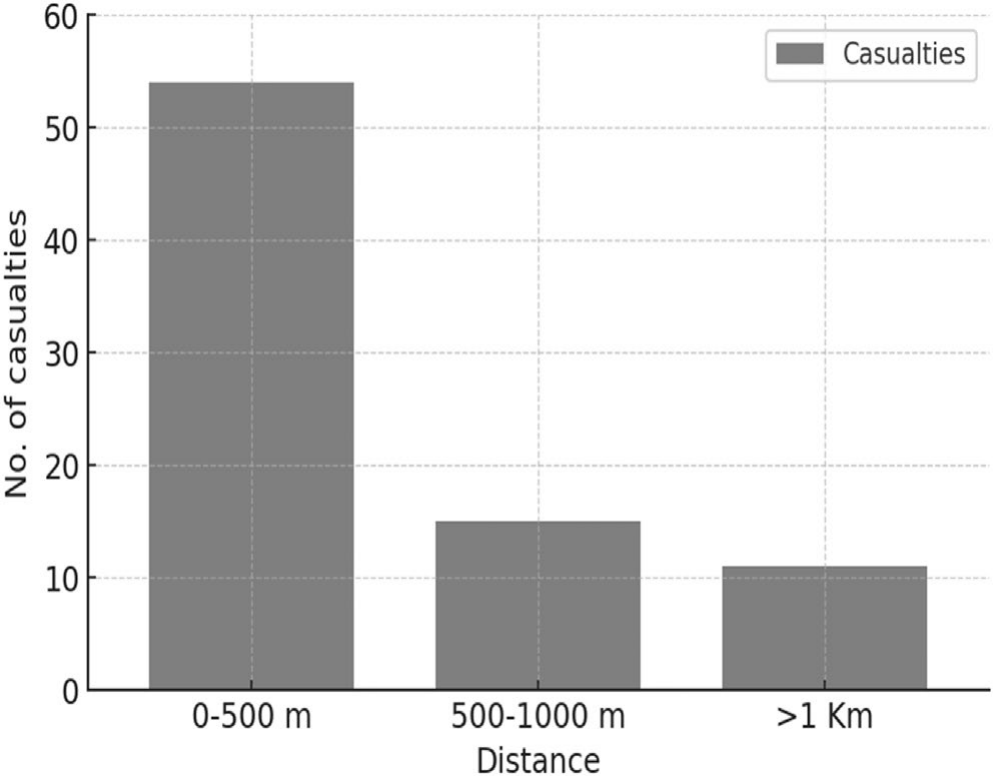
Distance of human casualties from the settlement areas into the forest.

**FIGURE 6:**
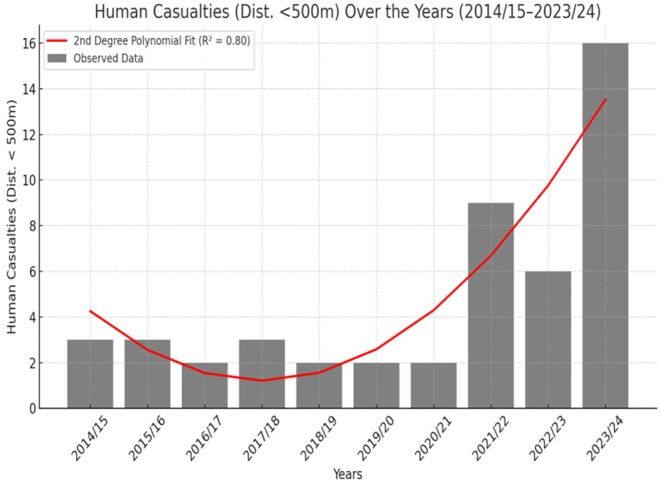
Years versus human casualties (Dist. < 500 m).

**FIGURE 7:**
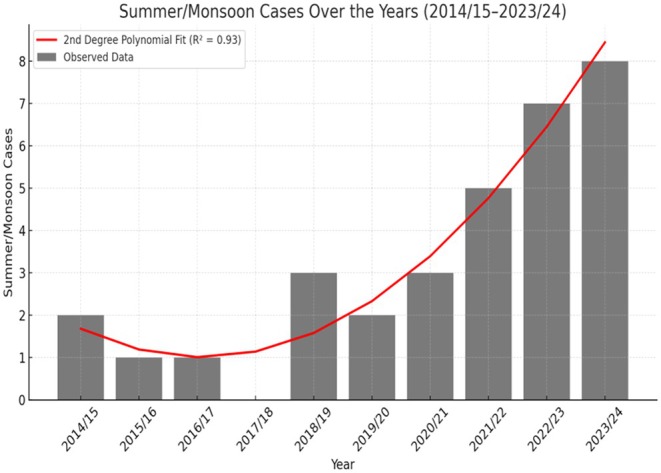
Human casualties: Years versus summer/monsoon season.

**FIGURE 8:**
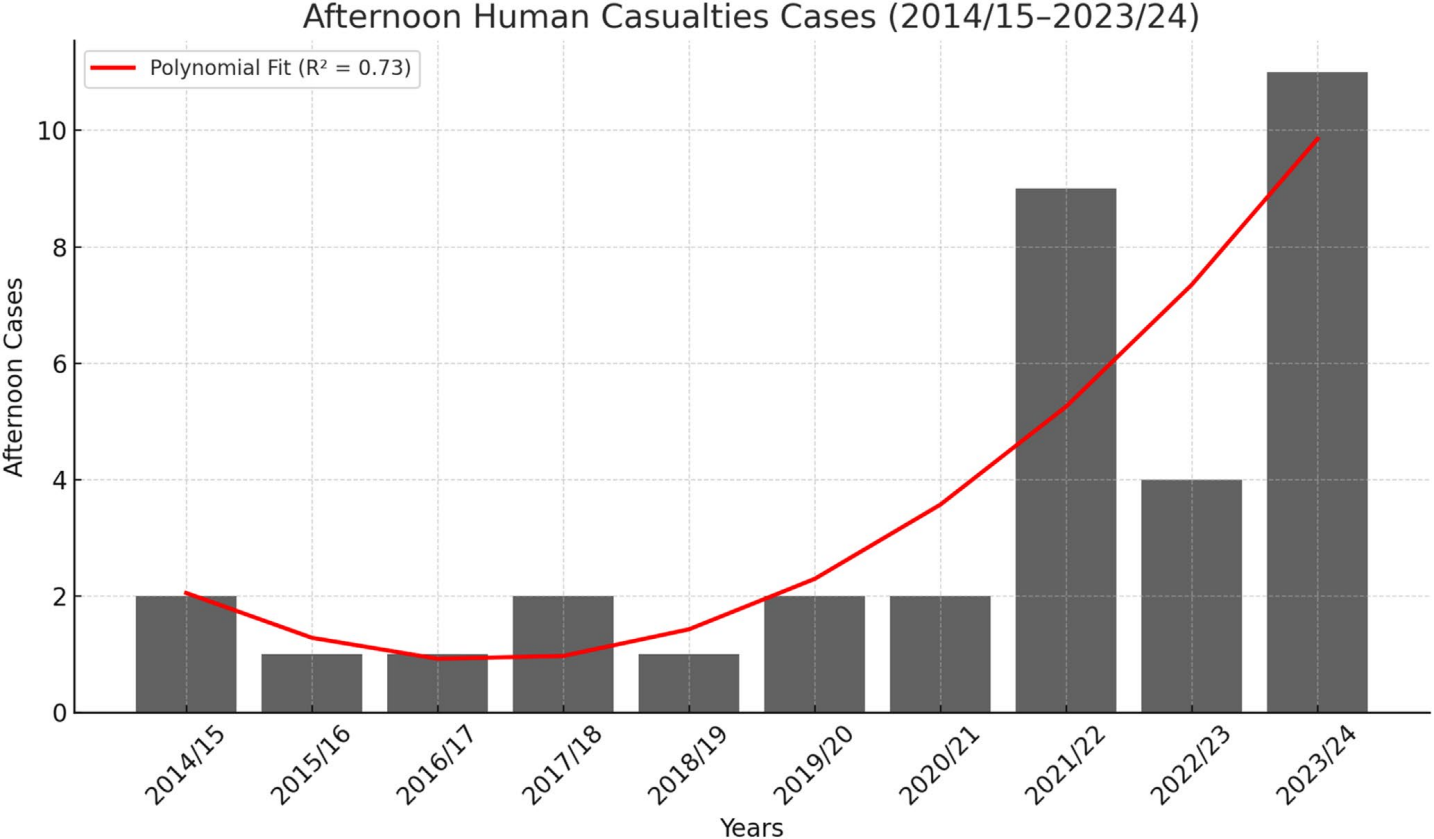
Human casualties with respect to afternoon cases.

**FIGURE 9:**
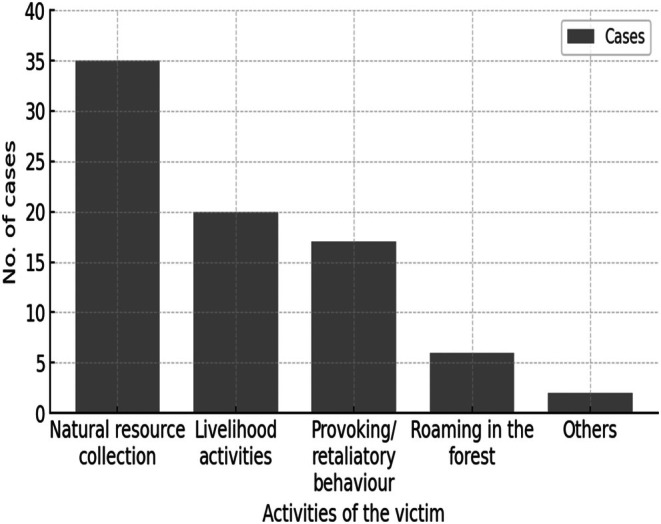
Activity of the victims during the HTC.

**FIGURE 10:**
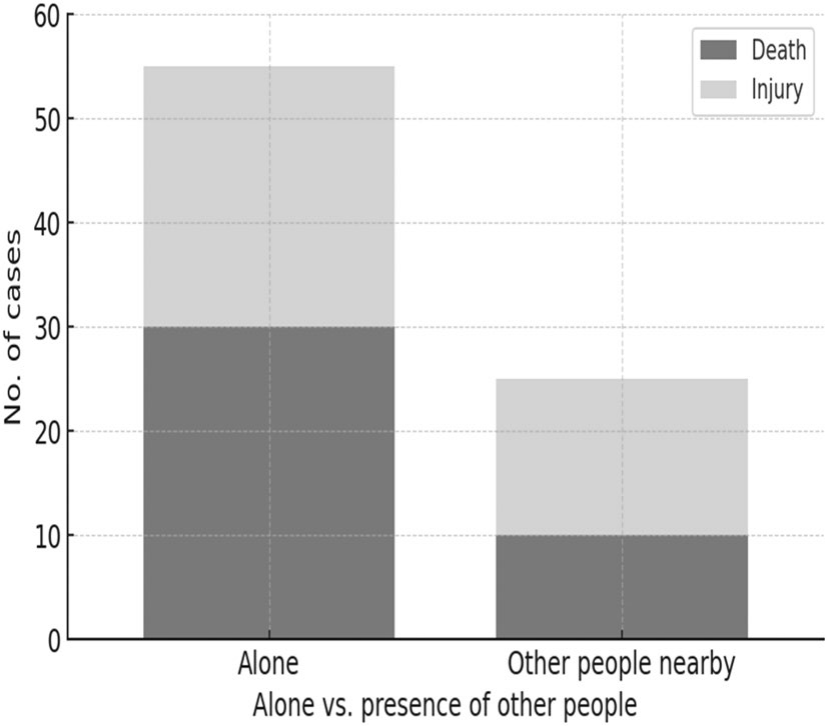
Comparison of human casualties: Alone versus presence of other people.

**FIGURE 11:**
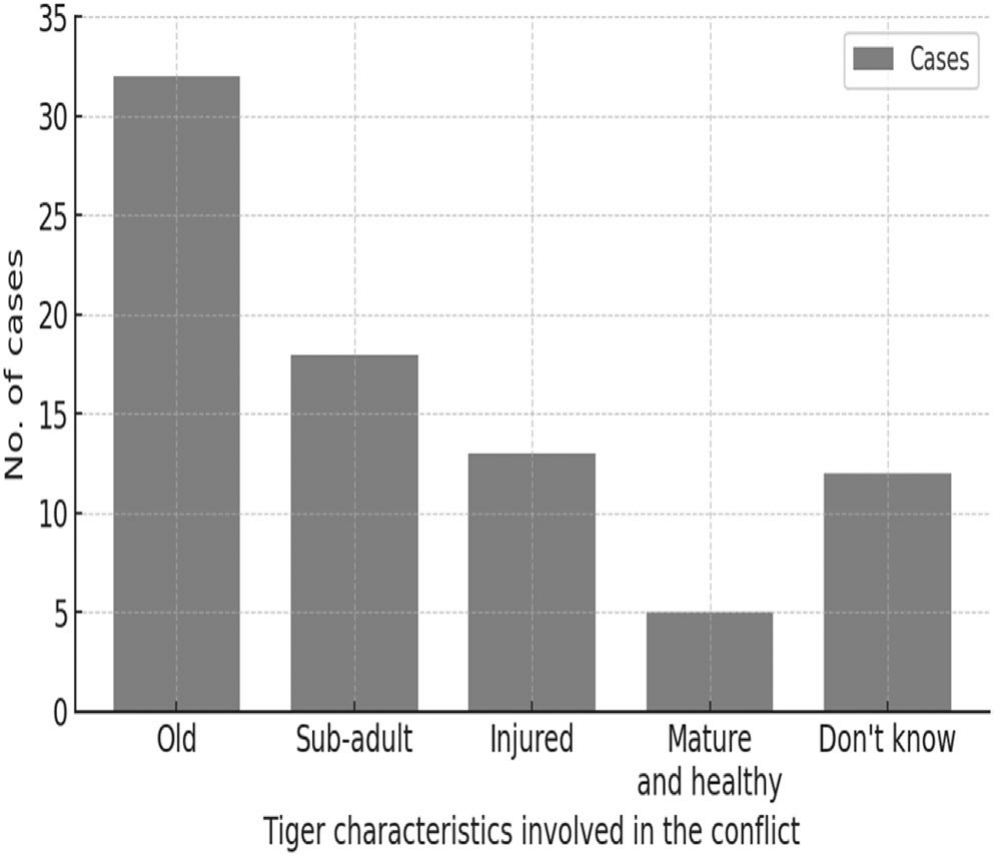
Tiger characteristics involved in the conflict.

**FIGURE 12:**
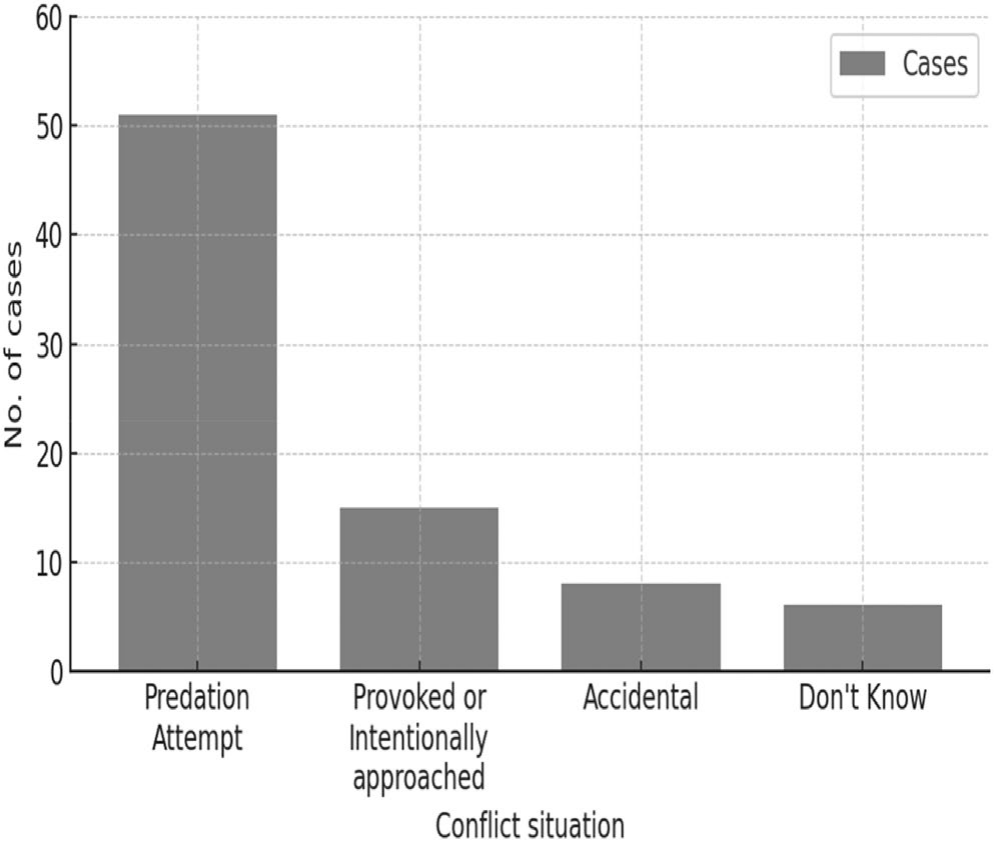
Conflict situation from the situation analysis.

This study summarized multiple factors associated with HTC by categorizing them into four broad domains: spatio‐temporal, human‐related, tiger‐related, and situational factors. Spatially, attacks were heavily concentrated in specific high‐risk zones such as Budhirapti, Madi, and Nandabhauju BZUC areas (Figure 4). Significant attacks happened in forest and near forest areas (*n* = 65, 81.25%), particularly within 500 m from human settlements (Figure 5) with a positive association (*R* ^2^ = 0.80, *y* = 0.3409*x* ^2^ – 2.7197*x* + 6.6333, *p* < 0.05) in the recent years (Figure 6). Casualties were higher inside PAs (i.e., BZs) with no occurrence outside from 2014/15 to 2018/19, but 15 cases (18.75%) were recorded from the more recent years, that is, 2020/21–2023/24, averaging 3.75 per year. Tiger attacks peaked during the summer/monsoon season (*n* = 32, 40%) followed by spring (*n* = 28, 35%), winter (*n* = 13, 16.25%) and autumn (*n* = 7, 8.75%), where it also showed a strong positive yearly trend (*R* ^2^ = 0.93, *y* = 0.1553*x* ^2^ – 0.9568*x* + 2.4833, *p* < 0.01; Figure 7). Attacks were most frequent in the afternoon (*n* = 41, 51.25%) followed by late morning (*n* = 16, 20%), early morning (*n* = 12, 15%), evening (*n* = 6, 7.5%) and night (*n* = 5, 6.25%), also with a significant upward trend (*R* ^2^ = 0.73, *p* < 0.05; Figure 8). Most tiger attacks occurred during resource collection (43.8%, *n* = 35; Figure 9) and most occurred when victims were alone at the time of encounter (68.8%, *n* = 55; Figure 10), with higher fatality rates (54.5%, *n* = 30). Tigers involved in conflicts were often old, injured, or sub‐adults, (Figure 11) and commonly employed ambush strategies, typically attacking from behind (*n* = 53, 66.25%). Of the tigers involved, 53.8% (*n* = 43) were known problematic tigers; the rest (46.3%, *n* = 37) had unknown histories. Over half of attacks by problem tigers were fatal (52.4%, *n* = 22). Tigers with prior human attack histories were associated with 57.9% (*n* = 11) fatalities, rising to 72.7% (*n* = 8) when victims were alone. Furthermore, situational factors such as predation attempts were dominant in many cases (Figure 12).

The statistical analysis in Table 3 shows the significance of several variables across all four domains. A strong association was found between proximity to settlements and the distribution of attacks (*χ* ^2^ = 42.32, *p* < 0.001), indicating that most incidents occur near human habitation. Temporal trends were also significant, with attacks varying across seasons (*χ* ^2^ = 23.43, *p* < 0.001) and times of day (*χ* ^2^ = 53.87, *p* < 0.001), reinforcing observed seasonal and daily activity patterns. Human behavior at the time of attack, particularly solitary presence and resource collection, was significantly associated with recorded attack patterns (*χ* ^2^ = 42.12, *p* < 0.01; *χ* ^2^ = 11.25, *p* = 0.01). Among tiger‐specific factors, the age‐class and health condition of the attacking tiger and the direction of attack (mostly from behind) were both highly significant (*χ* ^2^ = 27.37 and *χ* ^2^ = 48.8, *p* < 0.001). Conflict situations, especially predation‐driven incidents, also showed strong significance (*χ* ^2^ = 61.79, *p* < 0.001). While statistical details on victim awareness were limited, out of known cases, more than half of the victims (55.5%, *n* = 30) were aware of the conflict risk that may have influenced encounter outcomes.

**TABLE 3:** Factors associated with tiger attacks and their statistical significance.

Factor category	Variable	*χ* ^2^ value	df	*p*	Significant? (*p* < 0.05)
Spatial factors	Location of the attacks	*χ* ^2^ = 63.1	3	*p* < 0.001	Yes
	Distance of incidents from settlement (Figure 5)	*χ* ^2^ = 42.32	2	*p* < 0.001	Yes
Temporal factors	Seasonal variation in human casualties	*χ* ^2^ = 23.43	3	*p* < 0.001	Yes
	Time of day of human casualties	*χ* ^2^ = 53.87	4	*p* < 0.001	Yes
Human behavioral factors	Activity of victim during attack (Figure 9)	*χ* ^2^ = 42.12	4	*p* < 0.01	Yes
	Victim alone vs. in group (Figure 10)	*χ* ^2^ = 11.25	1	*p* = 0.01	Yes
Tiger‐related factors	Tiger age and condition (Figure 11)	*χ* ^2^ = 27.37	4	*p* < 0.001	Yes
	Attack position (front, back, side)	*χ* ^2^ = 48.8	2	*p* < 0.001	Yes
Situational factors	Type of conflict situation (Figure 12)	*χ* ^2^ = 61.79	3	*p* < 0.001	Yes

### 3.3 Perceived Preference on Mitigation Measures

**FIGURE 13:**
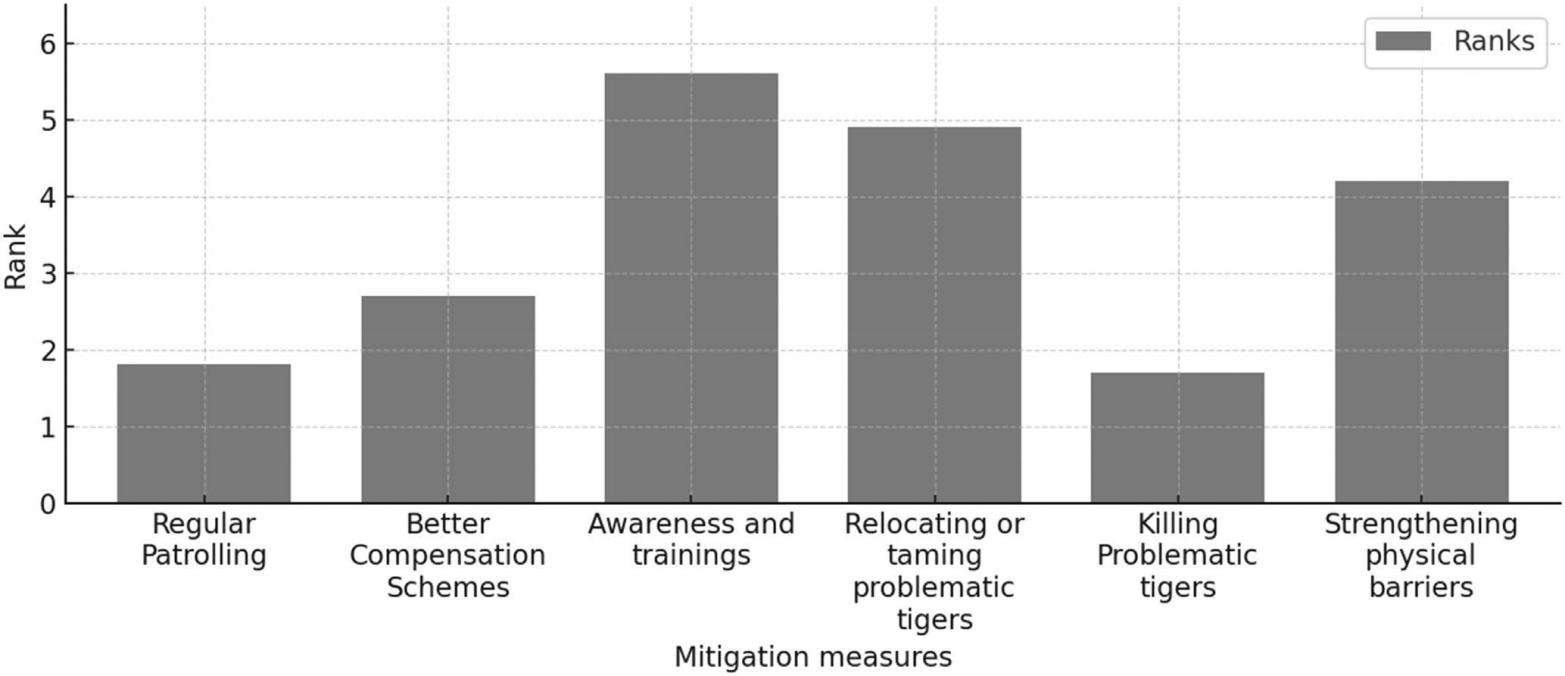
Perceived preference on mitigation measures based on ranks.

Friedman ANOVA showed significant differences (*χ* ^2^ = 311.42, df = 5, *p* < 0.001), with awareness/training ranked highest (5.56) and killing tigers lowest (1.75) (Figure 13). Despite attacks on family or livestock, 85% of respondents (*n* = 68) still supported tiger conservation in both cases.

**FIGURE 14:**
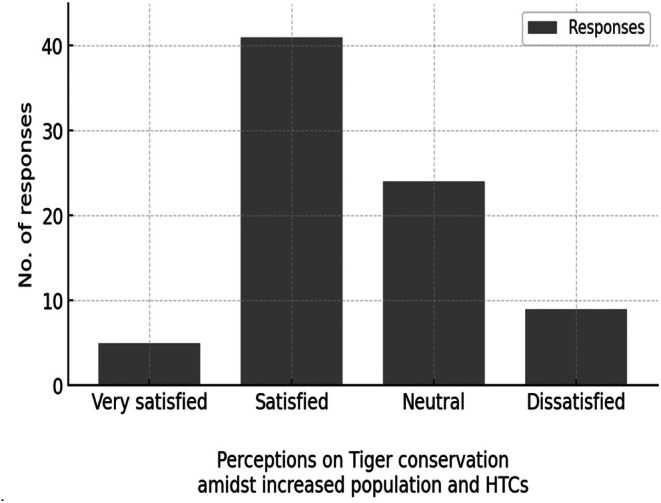
Perceptions on tiger conservation amidst increased population and HTCs.

**FIGURE 15:**
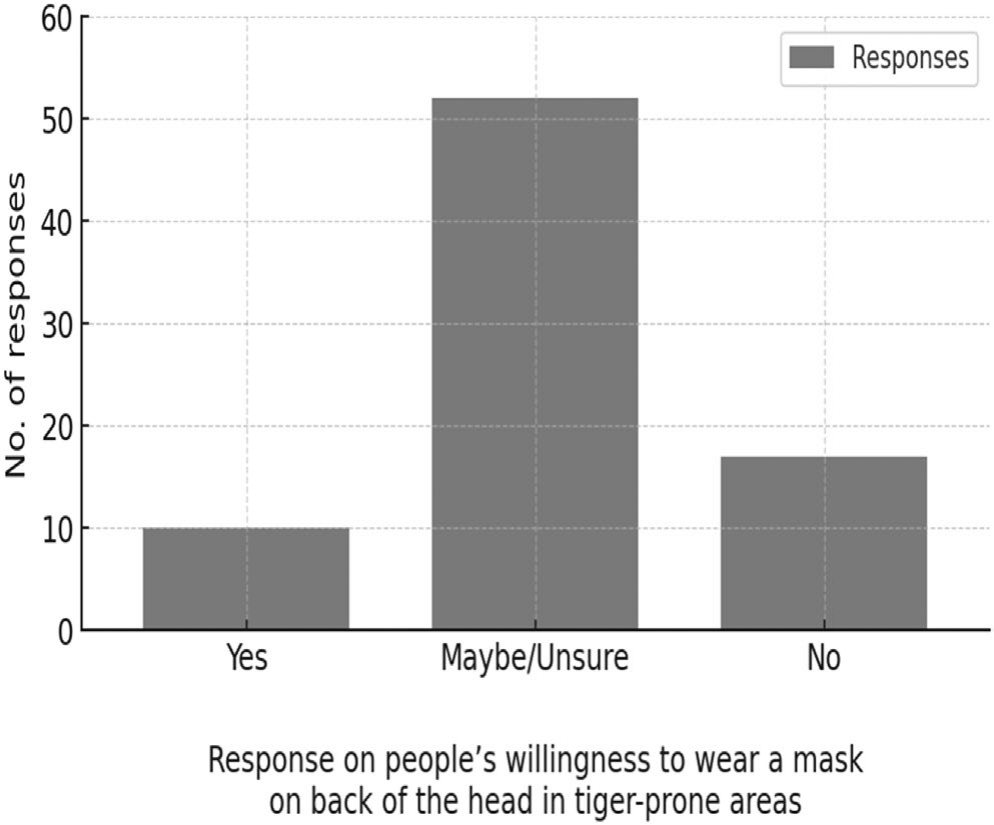
People's willingness to wear a mask on back of the head in tiger‐prone areas.

Significant differences were found in satisfaction levels with current HTC mitigation efforts (*χ* ^2^ = 38.7, df = 3, *p* < 0.001), with 51.25% (*n* = 41) expressing satisfaction. Similarly, 51.25% (*n* = 41) were optimistic about future coexistence (*χ* ^2^ = 59.4, df = 3, *p* < 0.001). Most respondents (67.5%, *n* = 54) held a positive view of tiger conservation despite recent increases in tiger numbers and HTCs (Figure 14). Compared to 5 years ago, 78.75% (*n* = 63) said their conservation sentiment remained unchanged. When asked about their willingness to try rear face masks, 80% (*n* = 64) showed some possibility, selecting “Maybe/Unsure” option (Figure 15).

## 4 Discussion

### 4.1 Status and Trend of Human Casualties

Between 2014/15 and 2023/24, 80 tiger attacks on humans were recorded in the study area, with most occurring in BZ of CNP. A substantial proportion of attacks resulted in fatalities, aligning with past studies (Nyhus and Tilson 2004; Dhanwatey et al. 2013; Dhungana et al. 2018). Over three‐fourths of victims were male, likely due to their greater involvement in forest‐related activities and limited education. This aligns with Silwal et al. (2017) but contrasts with Gurung et al. (2008), who reported more female victims from earlier decades (1980s to early 2000s) and incorporated nearly half of the incidents from the core areas of the park. Similarly, Nyhus and Tilson (2004) and Dhanwatey et al. (2013) also found that most victims of tiger attacks were males.

Human casualties from tiger attacks increased significantly over the study period, with annual averages rising from 4.6 (2014/15–2018/19) to 11.4 (2019/20–2023/24) (Figure 2). Gurung et al. (2008) reported 1.2 deaths/year (1979–1997), increasing to 7.2 (1998–2006), while Silwal et al. (2017) noted 6.1/year (2003–2013) and Dhungana et al. (2018) reported 7/year (2007–2014). These lower past averages may stem from several factors: Gurung et al. (2008) excluded injuries, and all three studies included core park areas where incidents often result from human trespassing, and are viewed as human faults which are rarely officially recorded. In contrast, our study focuses on BZs and adjacent forests, where attacks are more likely documented.

### 4.2 Factors Associated With Tiger Attacks

A positive association was observed between tiger population estimates and recorded human casualties during 2009–2022, in contrast to Lamichhane et al. (2018), possibly reflecting differences in study period and tiger population context. One possible explanation is that increasing tiger density may increase overlap with buffer zones and forest edges; however, this interpretation remains tentative because multiple ecological and human‐related factors may also contribute (Nyhus 2016). Although some suggest prey scarcity drives this behavior, recent prey base data (DNPWC and DFSC 2022) indicate otherwise as both tiger and prey populations have substantially grown. Research on the ECC of Bengal Tigers in the Chitwan–Parsa Complex (DNPWC 2020) estimates up to 175 tigers. As of 2022, the Chitwan–Parsa Complex supported 169 tigers, close to the previously estimated ecological carrying capacity of 175 tigers (DNPWC and DFSC 2022). This provides important conservation context; however, the observed rise in HTC should be interpreted cautiously as an association rather than evidence that proximity to ECC directly caused increased conflict as multiple ecological and human‐related factors may contribute.

Hotspot analysis (Figure 4) identified the Budhirapti BZUC region as an extremely high‐risk zone. Tall grasslands, especially during the monsoon, offer abundant livestock fodder and ideal tiger habitat (Figure 1), a finding supported by previous studies (Sanyal 1987; McDougal 1987; Mukherjee 2003; Gurung et al. 2008; Goodrich 2010; Bhattarai et al. 2017). These conditions may increase HTC risk, as tall grasses provide cover for tigers and may facilitate both accidental and opportunistic attacks. While Budhirapti BZUC had fewer tiger attacks historically, our study shows a recent rise. Earlier, Ayodhyapuri and Kalabanzar BZUCs were higher‐risk zones (Lamichhane et al. 2017). The increase may result from old, injured, or sub‐adult tigers moving toward human areas, aided by Budhirapti's forests, tall grasslands, scrub vegetation, and the consistent river water supply along three sides of the core area. Another high‐risk zone was identified in the Madi buffer zone, particularly around Panchpandav, Bagauda, and Rewa BZUCs supported by Lamichhane et al. (2017) prior to 2016. Here, human casualties were mainly linked to livestock herding. The Madi Valley's extensive forest edge adjoining CNP's dense core and its history as a livestock depredation hotspot further explain the elevated risk in this area. A third high‐risk zone was detected in Nandabhauju BZUC on the buffer zone's western side, mainly due to a rare 2016 incident where a sub‐adult tiger injured seven people in 1 day. Aside from this event, no cases were recorded, suggesting the area appears high‐risk on maps but does not consistently reflect true high risk. Very high and high‐risk areas surrounded the extremely high‐risk zones, while moderate risk zones were detected nearby, notably in the northern Madi Valley. Moderate risk area was especially evident in Patihani and Kerunga BZUCs within Kasara sector and the Nawalpur forest areas, raising concern due to the recent rise in cases.

This study shows that most incidents occurred within or near forests, mainly during natural resource collection (fodder, fuel wood, *niuro* or fiddlehead fern) or activities provoking wildlife. These findings align with global (Karanth and Madhusudan 2002; Nyhus and Tilson 2004) and regional studies (Gurung et al. 2008; Lamichhane et al. 2017; Dhungana et al. 2018) documenting similar patterns in CNP and surrounding areas. Our study found that most incidents occurred within 500 m of forested areas, consistent with Gurung et al. (2008) and Silwal et al. (2017), who reported most attacks within 1 km of forests, and Dhungana et al. (2018), who noted about two‐thirds of attacks fell within this range. The narrow, elongated core areas of CNP expand human–tiger interaction zones, increasing encounter risks. Our study shows a rising trend of attacks within 500 m of settlements over the past decade, with incidents outside the BZs emerging recently where no incidents have occurred in the first half of the study. This shift may reflect territorial pressures within a growing tiger population, which could increase the movement of old, injured, or sub‐adult tigers toward human settlements and thereby raise the possibility of HTC (Karanth and Madhusudan 2002; Nyhus 2016).

Human casualties from tiger attacks peaked significantly during the summer/monsoon season, with a rising trend during these periods suggesting potential for further increases (Figure 7). While research specifically on seasonal trends in tiger attacks is limited, Acharya et al. (2016) and Silwal et al. (2017) found no seasonal variation in CNP. In contrast, Kandel et al. (2023) noted higher attacks in spring and summer. Sundarbans studies reported seasonal peaks in April (Hendricks 1975; Das 2018), while Dhanwatey et al. (2013) found no seasonal trends in Tadoba‐Andhari Tiger Reserve. Seasonal variation in tiger attacks likely stems from ecological factors and human activities. Our study shows most incidents occur during summer/monsoon, when peak human activities like resource collection, grazing, and agriculture coincide with lush vegetation and increased tiger movement. Dense cover and abundant prey raise the chances of encounters, including ambushes (Karanth and Sunquist 2000; Wegge et al. 2009). Likewise high spring incidents align with frequent forest and agricultural activities, while fewer winter and autumn cases reflect reduced human movement as colder weather and harvest period limits human–tiger interactions.

Our study shows a significant variation in tiger attacks by time of the day, with most incidents occurring in the afternoon, followed by late morning. The observed upward trend indicates that this pattern may become more pronounced without timely intervention (Figure 8). This trend likely results from peak human activities such as collecting grasses, fodder, fuel wood, wild vegetables (e.g., *niuro*, mushrooms), and livestock herding during these hours. Nyhus and Tilson (2004) similarly reported afternoon attacks in Sumatra. Bhandari et al. (2020) found higher attack rates during afternoons, followed by mornings, in CNP (2014–2018). Paudel et al. (2024) observed almost all tiger attacks between 2019 and 2023 in BaNP‐BNP occurred during daytime, attributed to increased forest use, a pattern also noted by Fitzmaurice et al. (2021) for 2019 cases. Although tigers are mainly nocturnal, old, injured, or sub‐adult tigers may attack opportunistically at any time of day, especially when humans enter their territory (Nyhus 2016; Lamichhane 2018; Kolipaka 2018). This shift in behavior, driven by survival instincts, likely contributes to the tiger attacks observed in this study.

Our study found that many tiger incidents occurred while individuals were collecting forest products, such as grasses, fuel wood and wild vegetables (e.g., *niuro*). This is consistent with previous findings globally (Karanth and Madhusudan 2002; Nyhus and Tilson 2010) and locally where Gurung et al. (2008) found that most tiger attack victims in and around CNP were gathering fodder and similarly reported by Acharya et al. (2016), Dhungana et al. (2018), and Paudel et al. (2024).

Our study shows that over two‐thirds of conflict cases involved solitary victims, with higher lethality. Most incidents happening to lone individuals emphasize the protective role of group presence during tiger encounters. Solitary cases were more likely to be fatal, most resulting in death, compared to when others were present. This suggests that companions may deter attacks or aid intervention preventing fatalities. While limited, studies like Silwal et al. (2017) in CNP found group presence sometimes helped rescue victims and prevent fatalities. Several incidents from victim interviews highlight how companions have successfully deterred tiger attacks by shouting or using tools like stones, sticks, or sickles (Löe and Röskaft 2004; Mishra 2010; Silwal et al. 2017; Rice 2023). For instance, in Budhirapti BZUC, Balika Gurung, 55, was attacked from behind while returning from the forest after collecting grass and fuel wood. Her friends, who were ahead of her, reacted quickly, shouting and managing to flee the tiger away, ultimately saving her life. In another incident in the same BZUC, Bir Bahadur Tamang, 40, and his wife, Ujeli Tamang, were collecting forest resources when a tiger pounced on Bir Bahadur, biting into his head and neck like typical prey. Acting swiftly, Ujeli struck the tiger with a piece of wood, forcing it to retreat and saving her husband's life. Although the tiger had fully gripped his head, he survived.

Our study shows that old tigers were involved in the largest proportion of recorded conflict cases, followed by sub‐adults, injured, and healthy adults. This supports findings that aging tigers with declining abilities are more prone to livestock depredation and human encounters (Nyhus and Tilson 2004; Karanth and Gopal 2005; Gurung et al. 2008). Sub‐adults also contributed notably, likely due to hunting inexperience or dispersal challenges (Nyhus and Tilson 2004; Nyhus 2016; Lamichhane et al. 2017). Our study also shows that “problematic” animals, those with a history of attacking humans or livestock (Linnell 1999; Lamichhane et al. 2017; Shrestha et al. 2024), were involved in most of the cases, aligning with similar findings in CNP (Lamichhane et al. 2017). Fatal incidents were slightly more common among problematic tigers, with “man‐eaters,” those repeatedly attacking humans, making up about one‐fourth of cases with most ending in fatalities. Our study also highlights that attacks on lone individuals, especially by man‐eaters, often associated with severe outcomes, with three‐fourths being fatal.

Predation attempts accounted for about two‐thirds of HTC incidents, supported by the fact that most attacks were deliberate from behind, particularly by old, injured, or sub‐adult tigers reflecting their predatory instincts and driven by natural instincts of old, injured, or sub‐adult tigers displaced near settlements (Quigley and Herrero 2005; Gurung et al. 2008; Nyhus and Tilson 2010; Goodrich 2010; Nyhus 2016). About one‐fifth involved human provocation or accidental encounters (Goodrich et al. 2011; Dhanwatey et al. 2013; Nyhus 2016) whereas accidental encounters in some cases highlight the risks of human–tiger overlap in shared landscapes, supported by Goodrich et al. (2011); Nyhus (2016), reflecting the need for education, spatial planning, and avoiding high‐risk zones during peak tiger activity. Our study also shows that most victims were aware of potential risks but often underestimated the danger, likely due to limited knowledge of tiger threats. Others were unaware, highlighting the urgent need for educational campaigns about awareness and response to conflict risks.

### 4.3 Perceptions and Mitigation Measures

Our study highlights awareness and training as the most favored mitigation strategies, emphasizing that educating and empowering local communities remains central to effective wildlife conservation. This is consistent with global (Karanth and Madhusudan 2002; Löe and Röskaft 2004; Nyhus and Tilson 2010; Goodrich 2010; Dhanwatey et al. 2013; Nyhus 2016) and Nepal‐specific studies (Gurung et al. 2008; Dhungana et al. 2018; Lamichhane et al. 2018; Bhattarai et al. 2019; Sharma et al. 2024; Dhungana et al. 2024; Shrestha et al. 2024), which consistently advocate awareness and behavioral change to reduce conflict. The preference for relocating or taming problematic tigers as the second‐most favored measure reflects a reactive stance toward HTC incidents (Goodrich 2010). This indicates a growing concern over the increasing tiger population and dissatisfaction with indirect mitigation strategies. Proactive measures, such as reinforcing physical barriers like RCC and solar fences, were prioritized, corroborated by recent findings in CNP (Dhungana et al. 2024). Compensation schemes were regarded as secondary to direct mitigation, while regular patrolling and lethal control ranked lowest, likely due to concerns over resource limitations and a positive public perception of tigers despite conflicts.

This study highlights strong community support for tiger conservation despite HTC incidents, reflecting recognition of tigers' ecological and cultural value. While some concerns exist, overall, the community remains positive toward tiger conservation. The study also reveals mixed satisfaction with current HTC efforts, with most respondents satisfied but a substantial neutral group, suggesting room for improvement. Optimism for future tiger coexistence was high, though other majorities were neutral. There was strong support for tiger conservation despite an increase in the tiger population, with two‐thirds in favor and one‐third neutral, likely due to concerns over balancing conservation and safety. Most respondents maintained positive views over the past 5–10 years, though some reported declining support due to increased HTCs and unmet expectations. This raises concerns about the potential “bubble formation” in public sentiment where negative attitudes toward park management and long‐term conservation could emerge if current trends persist. Their attitudes, shaped by direct conflict experiences, are crucial for gauging public sentiment, highlighting the need to address their concerns to sustain long‐term support.

We analyzed tiger attack patterns and mitigation strategies, finding most attacks involved tigers pouncing from behind, often targeting the neck, similar to patterns reported in the Sundarbans and other areas as well (Karanth and Madhusudan 2002; Reza et al. 2002; Quigley and Herrero 2005; Montgomery 2009; Inskip et al. 2013). This led us to consider rear face masks as a mitigation option. While previous studies showed some success of it in tiger‐dense areas like the Sundarbans (Montgomery 2009; Das 2017), local responses were mixed: few believed masks would help, most were uncertain, and some were skeptical. Given less frequent forest entry compared to the Sundarbans, this method may still hold potential in our study area. When asked about using rear face masks, most respondents were uncertain about using them at present. However, their adoption could be promoted through targeted awareness campaigns in high‐conflict areas with frequent forest entry. Although not foolproof, even limited effectiveness could justify context‐specific testing and awareness‐based adoption in high‐conflict areas. Overall, the findings underscore the need for focused, research‐driven awareness programs to address gaps in understanding tiger behavior, human risk factors, problem individuals, and coexistence strategies, particularly in the context of increasing tiger populations and rising HTCs.

## 5 Conclusion

During 2014/15–2023/24, 80 human casualty cases from tiger attacks were recorded in the buffer zones of Chitwan and Parsa National Parks and surrounding forest areas. Among the study areas, casualties in CNP accounted for most cases with almost equal tiger attacks resulting in fatalities and injuries. Males accounted for over three‐fourths of the victims, likely due to their greater involvement in high‐risk activities such as forest resource collection, livestock herding, and retaliatory actions. Trend analysis shows a significant rise in human casualties from tiger attacks, with the average annual rate increasing from 4.6 cases (2014/15–2018/19) to 11.4 cases (2019/20–2023/24). A positive association was observed between tiger population estimates and recorded human casualties; however, this relationship should be treated as indicative rather than conclusive. Multiple concurrent factors including settlement expansion, forest‐resource dependence, and buffer zone population growth likely also contribute and cannot be disentangled from tiger population growth alone.

Density hotspot analysis identifies the Budhirapti BZUC region as an extremely high‐risk zone, likely due to its tall grasslands, abundant fodder, and suitability for tigers. The monsoon season, with sub‐adult, old, or injured tigers moving into human areas, may increase encounter opportunities. The Madi buffer zone remains high‐risk due to its forest edges and livestock herding. Moderate‐risk zones were detected in the northern Madi Valley and Nawalpur forest areas outside the park. Most HTC incidents occurred in forests during resource collection or activities provoking wildlife, with increasing attacks within 500 m of settlements over the past decade. These incidents peaked in summer/monsoon, followed by spring, possibly reflecting overlap among human activities, tiger movement, and prey availability. Attacks are most frequent in the afternoon and late morning, likely due to human presence and opportunistic behavior of old, injured, or sub‐adult tigers during the day. Most tiger incidents occurred during forest resource collection, such as gathering grass, fodder, or wild vegetables (*niuro*). Over two‐thirds of incidents involved individuals alone, highlighting increased vulnerability. Collective action in groups, such as shouting or using tools like sticks and sickles, proved protective in deterring tigers and often saved lives.

Older, injured, and sub‐adult tigers, driven by declining hunting abilities, inexperience, or territorial challenges, were primary contributors to attacks. Problematic tigers, including man‐eaters, were involved in most of the cases, with a high fatality rate, especially in attacks on solitary individuals. Attack patterns showed a preference for ambush from behind, typical of tiger predation, particularly among older and sub‐adult tigers. Situation analysis identified deliberate predation attempts, particularly by older, injured, or sub‐adult tigers near degraded habitats or human settlements, as the majority of cases. Provoking or intentionally approaching tigers also contributed to HTC cases, highlighting the avoidable nature of such conflicts. Despite prior awareness of conflict risks, victims often underestimated the threats, whereas some accidental encounters illustrate the risks of overlapping human–tiger spaces, which calls for the need for community education.

Awareness and training were the most preferred HTC mitigation strategies, followed by relocation of problem tigers and strengthening physical barriers. Limited support for lethal control reflects strong conservation values. Despite direct conflicts, community support for tiger conservation remains high, though neutrality toward park efforts suggests impending negativity. While optimism for coexistence persists, responsive management is needed. Skepticism toward rear‐face masks reveals knowledge gaps but has some promise. Targeted awareness and research‐driven strategies are critical for sustaining long‐term conflict mitigation.

We conclude that within the Chitwan–Parsa Complex, recorded HTC increased during a period of tiger population recovery. However, this pattern should be interpreted as associative rather than causal, and the findings should not be assumed to apply universally to other landscapes without context‐specific validation. Conflict dynamics in this landscape are shaped by multiple interacting factors, including tiger distribution, human activity near forests, settlement proximity, and forest‐resource dependence. Our study calls for targeted management of tigers and proactive measures to reduce vulnerabilities during human activities near tiger habitats in the Chitwan–Parsa complex. These findings are directly relevant to the Chitwan–Parsa landscape and may be informative for similar human‐dominated tiger landscapes in Nepal. Mitigation requires awareness, training on attack patterns, and cautious forest use. Strengthening barriers, relocating or taming problem tigers, and using rear‐face masks could help prevent attacks. While local conservation support remains, signs of “bubble formation” in public sentiment highlight the need for proactive management to foster coexistence.

## Author Contributions

**Aayush Shrestha:** conceptualization (lead), data curation (lead), formal analysis (lead), funding acquisition (equal), investigation (lead), methodology (lead), project administration (lead), resources (equal), software (lead), visualization (equal), writing – original draft (lead), writing – review and editing (lead). **Narendra Man Babu Pradhan:** conceptualization (equal), validation (equal), writing – review and editing (equal). **Naresh Subedi:** writing – review and editing (equal). **Bikram Shrestha:** investigation (supporting), supervision (supporting), validation (equal), writing – review and editing (equal). **Divesh Shrestha:** resources (lead), writing – review and editing (equal). **Mahesh Neupane:** resources (supporting), writing – review and editing (equal). **Krishna Prasad Dahal:** supervision (lead), validation (equal), writing – review and editing (equal).

## Funding

This work was supported by the National Trust for Nature Conservation–Biodiversity Conservation Center (NTNC‐BCC).

## Conflicts of Interest

The authors declare no conflicts of interest.

## Supporting information


**Data S1:** ece373920‐sup‐0001‐supinfo.xlsx.

## Data Availability

All the required data are uploaded as Supporting Information.
